# A podcast to teach medical humanities at medical school: a text-mining study of students’ lived experience

**DOI:** 10.1080/10872981.2024.2367823

**Published:** 2024-06-21

**Authors:** Emmanuel Roze, Christelle Nilles, Céline Louapre, Barbara Soumet-Leman, Marie-Christine Renaud, Agnès Dechartres, Cyril Atkinson-Clement

**Affiliations:** a DMU Neurosciences, Pitié-Salpêtrière Hospital, Paris, France; b Faculty of Medicine of Sorbonne University, INSERM, CNRS, Paris Brain Institute, Paris, France; c Neurology department, Hôpital Fondation Adolphe de Rothschild, Paris, France; d Plume De Harpie, Lamballe, France; e Département de Santé Publique, Sorbonne Université, INSERM, Institut Pierre Louis d’Épidémiologie et de Santé Publique, AP-HP, Hôpital Pitié-Salpêtrière, Paris, France; f Precision Imaging, School of Medicine, University of Nottingham, Nottingham, UK

**Keywords:** Podcasts, medical humanities, data mining, medical education, storytelling

## Abstract

The teaching of medical humanities is increasingly being integrated into medical school curricula. We developed a podcast called *Le Serment d’Augusta* (Augusta’s Oath), consisting of six episodes tackling hot topics in the modern world of healthcare related to the patient-doctor relationship, professionalism, and ethics. This podcast aimed to provide scientific content in an entertaining way, while promoting debate among medical students. The *Le Serment d’Augusta* podcast was proposed as one of the various optional modules included in the second- to fifth-year curriculum at the School of Medicine of Sorbonne University (Paris). We asked students to report their lived experience of listening to the podcast. We then used a text-mining approach focusing on two main aspects: i) students’ perspective of the use of this educational podcast to learn about medical humanities; ii) self-reported change in their perception of and knowledge about core elements of healthcare after listening to the podcast. 478 students were included. Students were grateful for the opportunity to participate in this teaching module. They greatly enjoyed this kind of learning tool and reported that it gave them autonomy in learning. They appreciated the content as well as the format, highlighting that the topics were related to the very essence of medical practice and that the numerous testimonies were of great added value. Listening to the podcast resulted in knowledge acquisition and significant change of perspective. These findings further support the use of podcasts in medical education, especially to teach medical humanities, and their implementation in the curriculum.

## Introduction

Recent societal factors such as the COVID-19 pandemic, an increased awareness of economic and climatic issues, and the #MeToo movement have contributed to a change in our perception of the outside world, our bodies, and what we expect from a healthcare relationship. In this context, patients and professional healthcare communities alike are showing a growing interest in open discussion about these burning issues [1,2]. Additionally, the combination of scientific evidence and experiential knowledge is highly valued by patients and their families, and there is an increasing awareness of the perceived lack of communication and emotional barriers created by some doctors [3–5] Indeed, developing an effective and empathetic relationship with the patient is at the heart of medical professions, and is known to improve quality of care and patient outcomes [6,7]. This refers to the medical humanities, which is a growing interdisciplinary field that explores the engagement and exchange between human experience and the world of medicine [8–10]. ‘Difficulty in supporting the humanities in medicine depends on perspectives of learning’ [11].

To address these topics, attractive teaching aids that encourage discussion and reflection among students, and that fit in with their lifestyle, could be of great interest [12]. Since the COVID-19 pandemic, the relationship between learning and mobility has changed in health education [13]. Students seem to want more autonomy and control over their learning, and some of them believe that there are ways of learning other than face-to-face, in line with their perception of the working world. Among alternative forms of media outlets and digital forms that can serve this purpose, podcasts are popular educational tools [14–16]. Because of their accessibility, flexibility and entertaining format [14,17–19], they are particularly adapted to our post COVID-19 world [13,18,20]. They are also easy to share via messaging, or on social media, which increases impact, and can suit everyone’s learning pace [19,21]. Learning through podcast may be seen as a ‘passive’ way of learning [19], but a more active component can be added (e.g., post-listening individual written comments or group debate sessions), to optimize information retention [22,23]. Finally, learning through podcasts intrinsically increases students’ control and autonomy, which are well known determinants of motivation to learn according to the dynamic model of motivation [24]. Medical education podcasts have been increasingly used in the past decade [25–28], although their effectiveness and optimal use is still poorly known [11,13,14]. A scoping review of podcast use in medical education found that 55–90% of listeners reported changing their practice after listening to a podcast [29]. One such podcast entitled ‘Not Otherwise Specified’ hosted by Dr. Lisa Rosenbaum [30], covers medical humanities, the acquisition of which is critical for the practice of medicine [31].

In this context, we developed a podcast about medical humanities called *Le Serment d’Augusta* (Augusta’s Oath) in partnership with *Binge Audio* (an independent French podcast production company). The title refers to Augusta Klumpke who was the first woman to obtain a residency in Paris; she went on to become a researcher and a pioneer in the field of neurology, especially in neurorehabilitation therapy [32].

The aim of this study was to analyze the participating students’ lived and subjective experience of listening to the podcast using a text-mining approach [33], focusing on two aspects: i) the students’ perspective about the use of this podcast as a tool to learn medical humanities; and ii) how listening to our podcast could change their perception and knowledge of core elements of healthcare in our modern world.

## Methods

### Study approval and consent

The study was approved by the Research Ethics Committee of Sorbonne University. The work was carried out in accordance with the declaration of Helsinki. All acquired data were anonymized and written consent was obtained from the participants.

### Study design and population

The study was based on an online survey and was conducted from October 2022 to June 2023 at the Medical School of Sorbonne University, Paris, France. Second- to fifth-year medical students were invited to voluntarily participate in the medical education program *Le Serment d’Augusta*, proposed as one of the various optional modules included in the curriculum https://www.binge.audio/actualites/le-serment-daugusta. This pilot learning experience consisted of listening to six podcast episodes over the 9-month period of the academic year. To validate having completed the module, the students had to: i) give their written opinion about the topics before listening to the episodes; ii) write a comment for each episode (Questions 1 to 6 – Q1 to Q6) explaining whether and how listening to the podcast changed their mind or not; and iii) give their general opinion about the podcast as a teaching medium (Question 7 – Q7). The students were asked to produce at least 100 words per question and not to use any idioms, acronyms, or implicit references. They were informed in advance that the content of their comments would not influence validation of the test. Additional data about the participants were collected: age, sex, presence of healthcare professionals among close relatives (yes/no), and perceived level of material comfort (very bad, bad, medium, good, very good). After anonymization of the data, responses to the questions (Q1-Q7) were analyzed for research purposes.

### Intervention

The podcast *Le Serment d’Augusta* aims to teach medical humanities and doctor/patient relationships to medical students. The ‘*Serment’* evokes the Hippocratic Oath, suggesting that medical students can reinvent their future commitment as medical doctors. The goal was to provide reliable, scientific content using an innovative format while promoting debate among medical trainees. In addition, it was designed to allow many people to express and share different opinions on sensitive subjects. It is a free resource, available to all students and to the general public, and free from advertising. It comprises one prologue and six episodes, each lasting around 50 minutes. Episode 1 (‘I will think of bodies outside the norm’) uses the paradigm of ‘fatphobia’ to discuss representation biases among healthcare professionals, which often result in discrimination. Episode 2 (‘I will cultivate a benevolent and inclusive solidarity’) narrates experiences of solidarity among healthcare professionals (especially during the COVID-19 pandemic) to decipher the systemic and cultural factors likely to influence solidarity and have consequences on quality of care. Episode 3 (‘I will seek consent actively and at each instant’) uses the paradigm of gynecological violence to explore the various aspects of consent to care in clinical practice. Episode 4 (‘I will take care of myself to take care of others’) focuses on mental health issues in healthcare professionals, especially the consequences on quality of care and the difficulties that may prevent healthcare professionals from asking for help. Episode 5 (‘I will look a little further for the truth’) questions the concept of scientific truth with examples from the COVID-19 pandemic. Episode 6 (‘I will look after those I cannot see’) focuses on healthcare for prisoners, emphasizing that this population is disadvantaged in terms of health and requires an active effort from healthcare professionals to compensate for disparity.

### Data preprocessing and analyses

**Figure 1.**
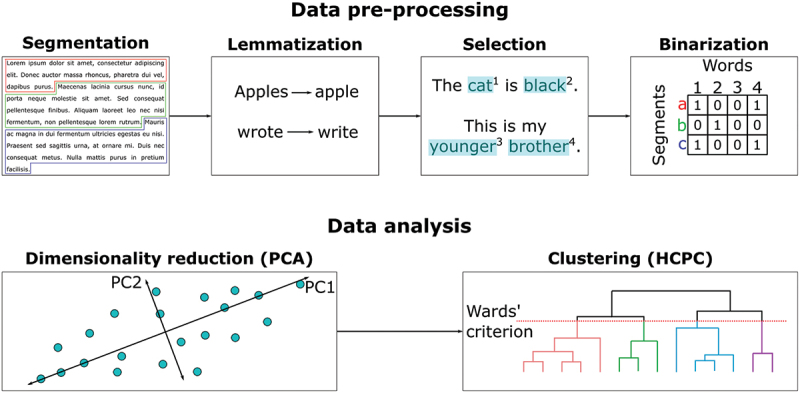
Data analysis pipeline. The data were first preprocessed (i.e., segmentation, lemmatization, word selection, binarization) and then analyzed (i.e., PCA, HCPC).

The analysis of the podcast as a whole (Q7) and of each episode (Q1-Q6) required the following steps (see Figure 1):

Step 1: each student commentary was decomposed into smaller units called ‘segments’ of approximately 40 words by respecting, when possible, sentences and punctuation;
Step 2: each segment was lemmatized, which means that each word was transformed into its most basic form (no conjugation, no agreement)
Step 3: words were selected based on the following criteria: they had to be composed of at least three letters, used by at least 5% of the participants, and be part of the following groups: adjectives, adpositions, adverbs, interjections, nouns, proper nouns, verbs (as tagged by the spacyr package [18]; https://spacy.io/) Punctuation, numbers, symbols or ‘stop words’ (i.e., words of very high frequency and nonspecific meaning, e.g., ‘the’, ‘this’, ‘it’ etc.) were not selected.
Step 4: a binary matrix was built for each corpus (i.e., all segments extracted for one specific question), which included all text segments as rows and all selected words as columns. The number ‘1’ was used when a word was used in a segment, while ‘0’ was used when a word was missing in a segment.
Step 5: we applied Principal Component Analysis (PCA) to the raw binary matrices to reduce the data complexity to only keep the most important information (i.e., the first five dimensions).
Step 6: finally, we applied a Hierarchical Clustering on Principal Components (HCPC). The optimal number of clusters was determined using the Ward’s criterion (i.e., threshold based on the selection of the option which leads to the highest relative loss of inertia)

To describe the clusters, we used a combination of v.test on the words (i.e., identify if a given word was more likely to be found in a specific cluster in comparison with the whole corpus) and a specificity test on the segments (described in Appendix 1). The 10 segments of each cluster with the lowest score were considered the most specific and they were used to interpret the meaning of the cluster. Lastly, we computed the proportion of each cluster found in the comments of each participant to investigate any statistical associations with socio-demographic variables using Anova or linear regression models, respectively, for categorical and numerical variables. The threshold for significance was set at *p* ≤ 0.05 following the Bonferroni correction.

All the data preprocessing and analyses of the raw textual data (i.e., in French; translation into English was only done to report results) were performed using the free software R [34].

## Results

### Main characteristics of the medical students

Among a potential population of 1807 students (70.7% women [*n* = 1277]), mean age 22.2 years (SD = 2.6), 478 students were included in the study. The mean age of the participants was 21.5 years (SD = 1.9), with an over-representation of women (80.9% [*n* = 365]). Most participants claimed to live in good or very good conditions (very good: 35.4% [*n* = 158], good: 46.6% [*n* = 208], medium: 15.2% [*n* = 68], bad: 2.2% [*n* = 10], very bad: 0.4% [*n* = 2]). Most participants did not have healthcare professionals among their close relatives (no: 69.6% [*n* = 312].

There was no significant association between response patterns and the students’ characteristics.

### General opinion about the podcast

Our analysis showed that the podcasts were appreciated both for their content because the topics touch on the essence of medicine and a doctor’s values, and for their format because of the use of audio files and testimonials. The students described the podcast as being an ‘interesting’, ‘playful’, and ‘easy’ teaching tool. They were grateful to have had access to the podcast and emphasized that this type of medium allows autonomy of learning (e.g., pausing; double-tasking). With regard to the module validation method, they reported that they enjoyed making comments because it encouraged them to reflect on the topic covered by the episode and to see how their thinking evolved. Some students provided recommendations for a better mode of validation.

### Episodes 1–6

Students gained awareness about representation bias and the normative body in medicine, and about the role of psychological factors in obesity in Episode 1 (‘Representation bias and discrimination in healthcare’). They became aware of the extent of fatphobia and prejudice against overweight people in the healthcare field, and of the negative consequences this has on the quality of care. They learned how to tackle this issue by creating a trusting, empathetic and inclusive relationship, and how to better address the issue of weight during a consultation.

In Episode 2 (‘Solidarity among healthcare professionals’), most of the students appreciated that the traditional culture of French medical schools was about to change by eliminating the discriminatory component – in particular sexist stereotypes – to become more respectful and inclusive. They realized the importance of solidarity and team spirit among healthcare professionals to ensure good quality of care.

Students learned what consent to healthcare was, how important it was, and how to obtain consent in clinical practice in Episode 3 (‘Consent to healthcare)’. The focus was on the fact that consent should be ‘explicit’ and that obtaining consent to healthcare is an active, ongoing process that depends essentially on a relationship of trust between patient and doctor. The students realized that progress needed to be made on the issue of consent, especially in the context of gynecological examinations.

In Episode 4 (‘Mental health of healthcare professionals‘), they also realized that mental health issues were very common among medical students and healthcare professionals, due to healthcare professionals’ particular vulnerability to psychological difficulties and the pressure they face to keep working. They stated they would pay special attention to the difficulties their colleagues might encounter. They became aware that caring for themselves was crucial and that they should not be ashamed to ask for help. Some students mentioned that they felt less lonely/isolated about their psychological problems after listening to the episode.

After listening to Episode 5 (‘Scientific truth’), students learned that scientific truth is by nature ephemeral and fragile, and realized the critical influence of social networks on the transmission and dissemination of scientific information, with the risk of reading ‘fake news’ and/or being trapped in a filter bubble. They became more eager to stay well-informed throughout their careers from primary-sourced information on reputable websites, so as to provide optimal care to their patients and respond to their questions appropriately.

Finally, they gained a new perspective on the issue of people excluded from the healthcare system in Episode 6 (‘Populations with loss of healthcare opportunities’). They learned about the need to make special efforts to provide appropriate care for disadvantaged or marginalized persons, particularly with regard to the quality of human interaction. More specifically they became aware of the fact that prisoners can be deprived of healthcare, especially in the context of psychiatric disorders and chronic illnesses, and that imprisonment itself is a significant ‘cause’ of morbidity and mortality.

**Figure 2.**
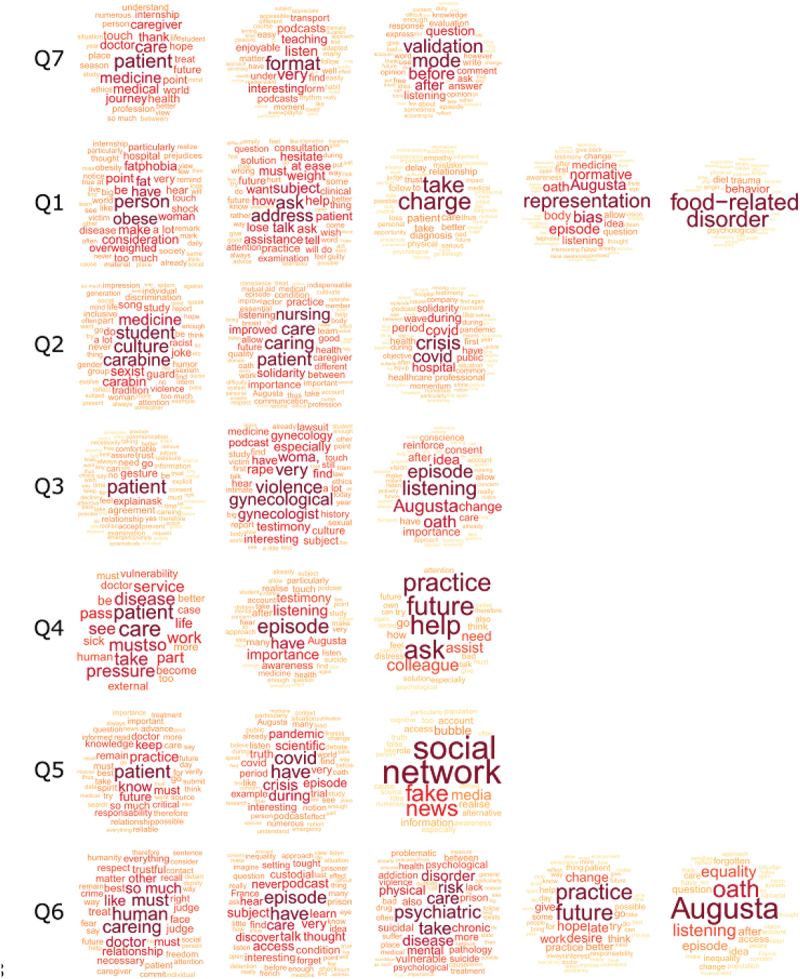
Word clouds displaying significant words obtained for each cluster of questions Q1-Q7. Only words with a significant positive v.Test are shown (i.e., indicating over-representation within the cluster compared with other clusters). The size, color, and location of the words depend on their importance (i.e., a higher v.Test score is represented by a larger word, in red and centered).

The main points related to opinions about each episode (1 to 6; Question 1 to 6) are summarized in Figure 2 and Boxes 1–7 whereas detailed analysis is provided as a supplementary result (Appendix 2).

## Discussion

This text-mining analysis of students’ lived experience of listening to the six-episode teaching podcast *Le Serment d’Augusta*, shows that they were grateful to have had the opportunity to participate in the teaching module. They greatly appreciated the podcast format as a learning tool and explained that it gave them better control of their learning with the possibility of double-tasking (such as listening during commute times) and self-pace learning. As well as the format, they also enjoyed the content emphasizing that the topics were related to the very essence of medical practice and that the multiple testimonies were of great added value. Listening to the podcasts resulted in increased awareness about topics related to medical humanities and changed their perspective. These findings further support the use of podcasts in medical education, especially to teach medical humanities and their implementation in the curriculum.

### A podcast to teach medical humanities

To the best of our knowledge, *Le Serment d’Augusta* is the first podcast about medical humanities that has been formally included in a medical student curriculum. Listening to our podcast was optional at the time of the study but has since become part of the curriculum for all students after this positive pilot experience.

Among the medical podcasts currently available, only a small proportion are produced by universities or professional societies, and advertising (not present in our podcast) is common [35]. Although rare, a few podcasts summarizing or presenting medical and scientific data have been produced and selectively target medical students. For example, two podcasts, containing key points from lectures and morning reports, have been developed for internal medicine residents in the USA [36,37]. Listeners found that it was an effective educational supplement that strengthened the links between the faculty and residents [36,37]. Interestingly, the Mayo Clinic has made podcasting part of their academic portfolios [38]. Other types of podcasts discussing equity in care and structural racism with a focus on medicine are available on the Internet but are not included in a curriculum [17]. These podcasts are designed to increase the listener’s awareness, and start important conversations on social media [17], which is a first step towards changing practices. The availability of *Le Serment d’Augusta* to all health professionals and the general public aims to encourage open debate and develop a sense of community. We think that integrating such a podcast into the curriculum is a critical step to prioritize topics related to the patient-doctor relationship, and underlines the importance of medical humanities. Our study showed that the topics covered in *Le Serment d’Augusta* were highly relevant to the students. With these podcasts we aimed to fill a gap in the current clinical learning curriculum in France by encouraging students to reflect on and debate topics that are not often discussed in conventional training [12]. The teaching of medical humanities is an increasingly important subject in the medical school curriculum [11,39,40] in an overall context of heightened societal awareness of the importance of diversity and inclusion which is especially relevant in the quality of the healthcare professional/patient relationship. Such teaching can improve medical professionalism, critical thinking, clinical judgement, empathy, communication, and the appreciation that patients’ problems go beyond the biology and consequently impact healthcare outcomes. It can also help prepare students for their future career and their adaptation to work-related stress [41,42]. While students value the existence of dedicated teaching of the humanities as a complement to biomedical teaching [43,44], efforts to widely integrate this teaching into the curriculum remain limited in France, partly due to time constraints and work overload. To achieve greater inclusion in the curriculum, medical humanities should ideally be incorporated into the curriculum through the lens of biomedical science and clinical practice [45,46]. A podcast thus represents a well-suited option considering the potential limitations and characteristics of the optimal learning content. Other educational strategies could be relevant to teach medical humanities such as (but not limited to) problem-based learning, team-based learning, standardized patients exercise, special interest groups, and interactions with a patient-as-a teacher [2,47–49]. These approaches would likely require more curricular time and space but may also foster student engagement.

### Teaching levers and episode duration

Podcasting allows the communication of information for medical students alongside entertainments program [21]. The positive perception of students and the quality of learning achievement after listening to our podcast could be related to the specific pedagogical levers we applied to ensure effectiveness and student involvement/satisfaction: i) we used powerful storytelling techniques sustained by a narrative structure and strong testimonies, along with immersive soundscape and music. Storytelling is an effective tool to teach medical topics [50] and can also help medical students cope with the challenges of healthcare relationships during their first field experiences [51]. ii) we approached topics from a transdisciplinary perspective so that students can listen to interviews and/or quotes of academics from both medical and social sciences. This transdisciplinary perspective can offer unique opportunities for meaningful connections to be made between the humanities and clinical experience [52]; iii) we included patient experiential knowledge to foster empathy towards patients [53,54]; iv) we integrated testimonies from medical students and young physicians, helping the audience to identify with their stories [55,56]. To make full use of these teaching levers, we chose a format of around 50 minutes per episode. This duration is unusually long compared to most medical education podcasts which are usually of a recommended duration of 15-20mn [14,57]. When podcasts are used to help memorize lectures, medical students have been found to report that even a duration as short as 3–5 minutes could be optimal [58]. However, our students did not comment negatively about the duration of the episodes. Finally, it is worth noting that we met the suggested consensus quality criteria for podcasts used in medical education comprising easy access, citing references and disclosing conflicts of interest, clearly identifying authors, and making a clear distinction between facts and opinions [22]. Overall, our approach aims to develop personal responsibility for learning: the students were expected to listen to each episode, and we hoped the podcast would stimulate reflection after presenting a variety of viewpoints and food for thought. However, we cannot be certain that they actually wrote their comments themselves and did not use generative artificial intelligence [59]. A reasonable option to partially address this issue would be to supplement podcast listening and written comments with group debates.

### Strengths and limitations

The study was innovative in that we used a text-mining approach to analyze the students’ opinions of their teaching program through free written expression i) with no *a priori*, ii) with the emphasis on ‘natural language’ data to evaluate the students lived experience, and iii) analyzing a large volume of textual data reflecting the experience of 478 students. Data mining is a qualitative or hybrid approach [60] since i) it is based on qualitative data, ii) it often requires having a deep knowledge of the corpus, and iii) it was found to produce results mostly comparable to those obtained with manual approaches [61]. Data mining is relevant for many different objectives including summarizing texts, extracting terminology, identifying important topics, assessing the emotional tone of a text, or reviewing the literature [62]. Our team already used data mining to identify the daily-living experience of teenagers suffering from Tourette syndrome [63]. In education [33,64,65], data mining can be useful for student evaluation [66], or for determining student satisfaction with a teaching module [67,68]. Text-mining has also been used to provide a better understanding of curricula offered by many higher education institutions thereby helping students decide which one they want to join [69]. This kind of approach has already been used to identify and correct aspects which make learning difficult [70], use sentiment analysis to enhance pedagogy [71], determine factors that increase student satisfaction [72], and to explore the impact of socialization on the formation of students professional identity [73]. In our study, it enabled us to identify any difficulties or disagreement about the form or content of the podcasts.

Some limitations deserve to be mentioned. First, generalizability of our results might be compromised because all the participants were from the same medical school in Paris and may not be representative of a general population of medical students in France. In addition, participation was optional, and we suspect that students with a keen interest in podcasts would have been more likely to participate in the teaching program, thereby introducing a potential recruitment bias. Second, the students’ responses might have been influenced by a social desirability bias or by the fear that their content could interfere with the validation process in spite of us making it clear from the beginning that the content of the response would not influence the validation process.

Although our questions were open-ended and the students could answer whether they had not changed their minds or not after listening to a podcast, the way we formulated the question may have skewed the results by inappropriately encouraging them to mention a change of mind. The absence of a control group and randomization prevented us from mitigating the selection and measurement biases.

## Conclusion

Our findings support the incorporation of podcasts in the medical curriculum to teach medical humanities. It is, however, important to keep in mind that most studies have assessed the educational value of podcasts based on subjective criteria and not on the consequences on patient care [14,29,74]. Further studies are needed to evaluate the impact of such a learning approach on the quality of care. In some cases, podcasting is a way for physicians to start a dialogue with patients [21,41]. A podcast – such as *Le Serment d’Augusta* - as part of the medical curriculum but also being freely available to the general public online, could foster an ongoing conversation between patients and physicians within the society.

**Box 1**: Opinions about learning with the podcast *Le Serment d’Augusta*. For topics corresponding to each cluster, this box shows a selection of representative segments chosen among the ten with the lowest (best) Specificity score of each cluster.

**Table:** 

**Q7 – Cluster 1 [47%]: Content and form of this podcast** *“In my opinion, these podcasts are about the very essence of medicine, about a doctor’s values, about the values that I wish to portray later in my career.” (score: 5.0, rank: 4)* *“I honestly think that these podcasts should not be optional because they deal with important subjects which are relevant to all future caregivers. Thank you so much!” (score: 5.1, rank: 5)* *“I think that I was more moved by the fact of hearing voices, testimonies, than if it had been a conventional lecture.” (score: 5.2, rank: 9)* **Q7 – Cluster 2 [27%]: Podcast as a way of teaching** *“I found that the teaching format was very good, it allows us to listen to the podcast while doing something else at the same time. In addition, the podcast deals with subjects that seem very important to me and that we rarely have the opportunity to discuss with the various teachers during our studies.” (score: 7.8, rank: 1)* *“I really enjoyed the format of this teaching, it’s easy to listen to, and the audio format in the form of a podcast is pleasant to follow. The subjects are interesting, well explained, and well-illustrated by the various testimonies.” (score: 7.9, rank: 4)* *“I found the format of the lessons very playful, and easy to use, especially during long journeys.” (score: 8.1, rank: 9)* **Q7 – Cluster 3 [26%]: Method for evaluating the students** *“I think that making comments is great, because it allows us to reflect and organize our ideas in a concise and precise way. This allows a kind of exchange after listening to professionals. It stimulates our thinking and allows us to actively contribute to the discussion.” (score: 4.9, rank: 9)* *“The evaluation mode is good since it allows us to see how our thinking evolves which can turn out to be completely different before and after listening.” (score: 4.9, rank: 5)* *“However, the validation mode may not be the most appropriate because writing 100 words before listening may be too much, we have not always been thinking about these issues previously and therefore we do not have very developed answers.” (score: 4.8, rank: 1)*

**Box 2**: Students” change of mind after listening to episode 1 (Representation biases and discrimination in healthcare). For topics corresponding to each cluster, this box shows a selection of representative segments chosen among the ten with the lowest (best) Specificity score of each cluster.

**Table:** 

**Q1 – Cluster 1 [42%]: Fatphobia in medicine** *“The ideas that already circulate in society about people with obesity continue to spread in the hospital, such as the idea that they are lazy, powerless, or even cowards.” (score: 5.7, rank: 4)* *“I am very surprised to see how fatphobia is present in healthcare. I thought that there was a good awareness about it in this environment, thanks to the knowledge and the ethics that we are taught.” (score: 5.8, rank: 5)* *“I had never realized that the thoughts I had about a person with obesity made me an actor of fatphobia.” (score: 5.8, rank: 7)* **Q1 – Cluster 2 [23%]: Considering weight issues in clinical practice** *“I learned that the best way to avoid being embarrassed in front of a patient is to simply ask him/her, tell him/her that we are not sure we have the right words, and to discuss with him/her the subjects that he/she is willing to address or not.” (score: 8.7, rank: 3)* *“I appreciated the advice of the resident, saying that it is also possible to ask the patient for help with examination, it seems to me that this is beneficial for both the patient and the doctor. Finally, I will take up the advice given to ask the patient if he/she would like us to discuss the question of his/her weight.” (score: 8.8, rank: 9)* **Q1 – Cluster 3 [23%]: How to tackle discrimination** *“It raises our awareness and shows us that we have to provide tailored care, remembering that the normative body is not reality and that our patients will all have different bodies. An inclusive practice is required, providing quality care regardless of appearance, culture or ethnicity.” (score: 4.3, rank: 2)* *“Biases in medicine have more repercussions than in other fields, since they are associated with medical errors and diagnostic delay that can be fatal. Thus, it is all the more important to work on the different biases that we may have, in order to offer patients the best possible prognosis.” (score: 4.4, rank: 4)* **Q1 – Cluster 4 [10%]: Changes about representation bias** *“Listening to this podcast did not really change my ideas about the issue of representational bias and the normative body in medicine, but it allowed me to reinforce my ideas and realize the importance of this bias in clinical practice.” (score: 10.2, rank: 1)* *“I think that listening to this podcast episode changed my ideas about the question of biases of representation and the normative body because it quite simply made me realize that I had such biases, and that many people have such biases.” (score: 10.4, rank: 7)* **Q1 – Cluster 5 [2%]: Psychological factors and obesity** “*I already had in mind the genetic predisposition at the origin of obesity but I did not realize the importance of trauma leading to eating disorders that must therefore be taken care of as a priority, before proposing diets which in most cases are ineffective*.” *(score:6.3, rank:2)* “*It is not necessary to make people with obesity lose weight at any cost, but it is necessary to understand their often complicated backgrounds, their possible eating disorders, their experience of violence, and we have to support them if they wish to lose weight in appropriate care structures*” *(score: 6.4, rank: 3)*

**Box 3**: Students’ change of mind after listening to episode 2 (Solidarity among healthcare professionals). For topics corresponding to each cluster, this box shows a selection of representative segments chosen among the ten with the lowest (best) Specificity score of each.

**Table:** 

**Q2 – Cluster 1 [48%]: Common traditional culture at the French medical school** *“It’s not normal that, as a medical student, we still accept the violent behavior of our peers, colleagues, and superiors because we have no other way out. Because it is a culture.”(score: 6.3, rank: 2)* *“It is also a tremendous source of hope to hear students who are concerned about the place of discrimination in our studies and our student life.” (score: 6.3, rank: 3)* *“It is very difficult to go to the end (of the medical studies). The “carabine” spirit is important in student life. Medical studies are long, difficult, and morally and physically demanding. This anti-stress side allows you to unwind.” (score: 6.3, rank: 4)* **Q2 – Cluster 2 [29%]: Solidarity and quality of care** *“You quickly realize during the rotations that getting to know each other within a team results in better care. This episode raises awareness of the importance of shared moments, for example during meals or breaks.” (score: 8.3, rank: 2)* *“By listening to the episode, we can really understand how solidarity between caregivers is of particular importance. It plays an essential role in patient care, of course, but also in the general functioning of the health system.” (score: 8.5, rank: 6)* **Q2 – Cluster 3 [23%]: Solidarity during the COVID-19 crisis** *“In times of crisis, we can see a surge of solidarity, which has surely made it possible to keep the public hospital running and to keep the caregivers on their feet during the COVID-19 crisis.” (score: 7.0, rank: 3)* *“Solidarity in the hospital and in the healthcare system seems more important than ever. I find it striking that health professionals have so much appreciated the incredible solidarity during the Covid crisis, which kept the hospital running.” (score: 7.1, rank: 8)*

**Box 4**: Students’ change of mind after listening to episode 3 (Consent to healthcare). For topics corresponding to each cluster, this box shows a selection of representative segments chosen among the ten with the lowest (best) Specificity score of each.

**Table:** 

**Q3– Cluster 1 [52%]: Consent to healthcare: *what and how*** *“We learn what is meant by consent, which must be explicit and not implicit, how important it is to create a trusting relationship with our patients, that you must take time to explain the why and how of a medical procedure and ask for permission to examine them beforehand.” (score: 8.0, rank: 1)* *“It can easily happen to take for granted the hesitant “yes” of a patient, who feels intimidated in front of a doctor and who does not dare to say “no”, or who would like more information, for a clear and definite “yes”” (score: 8.0, rank: 6)* **Q3– Cluster 2 [39%]: Gynecological violence** *“I realize through this podcast that the concept of consent, which seems simple to define, is actually more complicated to apply than what I thought – Especially, in the situation of gynecological emergencies where the rescue objective is prevailing and where the psychic trauma of women are largely neglected.” (score: 9.7, rank: 3)* *“I find it outrageous that there can still be violence, gynecological, medical, physical or verbal, in the practice of medicine (and not only). I found the point of the gynecologist on the importance very interesting, about paying attention to non-verbal behavior to appreciate consent.” (score: 9.8, rank: 10)* **Q3– Cluster 3 [9%]: Importance of consent to healthcare** *“Listening to the third episode of Le Serment d’Augusta made me realize the importance of obtaining patient consent in healthcare. In the future, I will have to be clear and precise when giving explanations to my patients.’ (score: 9.3, rank: 3)* *“Listening to this episode may have changed my ideas on the question of consent to healthcare: I was not aware of the importance of consent in the context of care for a patient’s psychological well-being.” (score: 9.4, rank: 7)*

**Box 5**: Students’ change of mind after listening to episode 4 (Mental health of healthcare professionals). For topics corresponding to each cluster, this box shows a selection of representative segments chosen among the ten with the lowest (best) Specificity score of each.

**Table:** 

**Q4 – Cluster 1 [62%]: Vulnerability of healthcare professionals** *“Perhaps we should change things and allow caregivers, even those who suffer from disabling illnesses, to be able to work differently, which is not often the case. When work is a vocation, stopping work is sometimes just as difficult as announcing an illness.” (score: 5.4, rank: 2)* *“At the same time, there is this pressure to hang on, as if the doctor could not be a patient, because he/she was a superman.” (score: 5.4, rank: 4)* *“Contact with death and illness is difficult to bear over the long term, which implies that working conditions in our professions are more difficult than for the general population.”(score: 5.4, rank: 6)* **Q4 – Cluster 2 [25%]: Caring for yourself** *“I found this fourth episode of Le Serment d’Augusta to be very rewarding and it opened my eyes to a lot of different aspects. The many testimonials from students and caregivers made me realize that almost everyone feels the same things at some point.” (score: 6.7, rank: 2)* *“Very touching episode, which highlights the difficulties we must face. Sometimes it”s even difficult to hear certain testimonies from our colleagues. The subject is introduced with a poignant story. Taking care of yourself must be a priority and this podcast makes it clear to us.’ (score: 6.8, rank: 9)* **Q4 – Cluster 3 [13%]: Asking for help** *“[…] or hide from the rest of the medical profession to ask for help, because obviously this should not be known. It’s just unfortunate. This is why, in my future practice, I will do my best to detect signs of distress in my colleagues, just as in myself.” (score: 4.5, rank: 1)* *“I liked that you suggested how to ask for help, and especially that you talked about the obstacles to asking for help. I have noted these resources and hope they will be of use to me in my future practice.” (score: 4.6, rank: 2)*

**Box 6**: Students’ change of mind after listening to episode 5 (Scientific truth). For topics corresponding to each cluster, this box shows a selection of representative segments chosen among the ten with the lowest (best) Specificity score of each.

**Table:** 

**Q5 – Cluster 1 [48%]: Continuous medical education** *“As a future caregiver, I would demonstrate scientific rigor and would always try to explain what I can to patients, even if it means telling them that I don’t know when I don’t know.” (score: 9.6, rank: 4)* *“This will impact me in my future practice, during which I will always be careful to properly inform my patients and keep myself as informed as possible, in order to know how to answer their questions in a serene manner.” (score: 9.6, rank: 5)* *“I think that this example should be kept in mind as a caregiver: we must always be aware of the limits of our own knowledge, not remain locked into our fixed beliefs, and take into account the existence of clinical situations that we may not have anticipated, in order to treat patients in the best possible way.”(score: 9.7, rank: 9)* **Q5 – Cluster 2 [42%]: Scientific truth during the COVID-19 crisis** *“This episode focuses a lot on the 2020 pandemic (and for good reasons) since in terms of information, misinformation and scientific truth in the medical world, this event has changed a lot of things. During the pandemic, scientific truth was undermined.” (score: 8.6, rank: 1)* *“Similarly, the thoughts about information provided to the general public during the Covid crisis are thoughts that I had already thought about a lot.” (score: 8.6, rank: 2)* *“Scientific truth depends on its time, and on the circumstances of its demonstration. We can see this very well in this episode with the different examples used, such as the discovery of the importance of hand washing for hygiene in medicine.” (score: 8.6, rank: 3)* **Q5 – Cluster 3 [10%]: Scientific truth and social networks** *“Also, the growing power of social networks, where people who spread fake news are in reality much more vocal and present than those who try to defend the scientific approach. The emotional bias plays a big role, the truths these people asserted with certainty were reassuring.” (score: 6.1, rank: 1)* *“Then, I think that it somehow raised my awareness regarding the importance of being attentive to the information that circulates around us, particularly in the media and on social networks.” (score: 6.3, rank: 9)* *“In addition, I also became aware of this filter bubble common to most social networks, which locks us up, without our realizing it, in a micro-society bringing together only points of view close to ours.” (score: 6.3, rank: 10)*

**Box 7**: Students’ change of mind after listening to episode 6 (Populations with loss of healthcare opportunities). For topics corresponding to each cluster, this box shows a selection of representative segments chosen among the ten with the lowest (best) Specificity score of each.

**Table:** 

**Q6 – Cluster 1 [36%]: Care equity** *“regardless of their age, gender, ethnic origin, and regardless of their actions, etc.” (score: 4.1, rank: 1)* *“It is therefore critical to reinject liveliness and humanity into our interpersonal relationships, in order to go beyond our societal bubble, to seek out those who are in a precarious situation on a daily basis, whether imprisoned or free, legal or illegal migrants.” (score: 4.3, rank: 6)* *“I was able to see through this example to what extent, even as a doctor thinking of acting and helping each person equally, we were capable of ‘giving up’ and treating an entire part of the population less well.“ (score: 4.4, rank: 8)* **Q6 – Cluster 2 [27%]: Health services in prison** *“It’s true that people who don’t make it to the hospital are invisible to us. They are forgotten by the society in general. I’ve never thought before about people in prison and how they get access to healthcare.” (score: 4.3, rank: 5)* *“With this podcast, I was able to understand the many problems of equal access to care, especially in prisons. I have to admit that I was surprised, shocked by the testimonies and that I have never been able before to imagine with precision the difficulties experienced by prisoners.” (score: 4.4, rank: 7)* **Q6 – Cluster 3 [23%]: loss of healthcare opportunity in prison** *“Imprisonment is an immense source of morbidity. Indeed, the prevalence of HIV is, for example, 3 to 4 times higher than in the general population, the dental health much worse and the frequency of psychiatric disorders and addiction much higher.” (score: 5.1, rank: 2)* *“The conditions in prison are very precarious, in terms of resources both for the inmates and the doctors who provide treatment there. This makes it very difficult to care for prisoners who have psychiatric disorders or chronic illnesses.” (score: 5.2, rank: 10)* **Q6 – Cluster 4 [11%]: influence on future practice** *“I don’t know if this episode will concretely change my future practice because I don’t think I will work with prisoners, but I think it is a very important societal subject.” (score: 6.5, rank: 3)* *“I hope to be able to take these lessons into account in my future practice to adapt to these different profiles and try to provide care to everyone without considering their social status or what they may have done in their life.” (score: 6.6, rank: 7)* **Q6 – Cluster 5 [3%]: new perspective on the forgotten of care** *“Listening to Episode 6 of Le Serment d’Augusta allowed me to reflect on the issues of equal access to care and allowed me to become aware of the populations that belong to the forgotten in care.” (score: 5.7, rank: 3)*

## Supplementary Material

Appendix 2_R2_Clean.docx
