# Supplementary material for: A podcast to teach medical humanities at medical school: a text-mining study of students’ lived experience
Source: Med Educ Online. 2024 Jun 21;29(1):2367823. doi: 10.1080/10872981.2024.2367823 (PMC11195461; doi:10.1080/10872981.2024.2367823)
Supplement: Appendix 2_R2_Clean.docx [file ZMEO_A_2367823_SM0273.docx]

**Appendix 2**

**General opinion about the podcast**

Question 7 (i.e., General opinion about the podcast) was decomposed into 1502 segments. Based on the HCPC, three different clusters were identified. The first cluster (47% of segments / 74% of the students; called “Content and form of the podcasts”) shows that the podcasts were appreciated both for their content because the topics touch on the essence of medicine and a doctor’s values, and for their format because of the use of audio files and testimonials. Of note, the word “thank” (v=5.2) was the fifth most specific word used in this cluster, illustrating that the students were grateful.

The second cluster (27% of segments / 68% of the students; called “Podcast as a way of teaching”) highlighted the advantages of the podcast format as a teaching tool (“format” [v=19.9], “teaching” [v=11.4], “podcasts” [v=11.3]), which the students described as being “interesting” (v=11.4), “playful” (v=9.3), and “easy” (v=7.7). The students enjoyed the podcast “very much” (“loved” [v=4.4], “enjoyed” [v=4.4], “very much” [v=5.5]) and emphasized that this type of medium allows autonomy of learning (e.g., pausing the podcast; double-tasking).

The third cluster (26% of segments / 63% of the student called “Method for evaluating the students”) was related to how the students who participated in the podcast teaching module were evaluated (“evaluating” [v=7.4], “validation” [v=19.4]). They reported that they enjoyed making comments because it encouraged them to reflect on the topic covered by the episode and to see how their thinking evolved. However, some students provided recommendations for a better mode of evaluation (“however” [v=6], “sometimes” [v=4.9], “difficult” [v=3.5]). In particular, some students found it difficult to discuss a topic before listening to an episode and would have preferred to give their opinion only after listening to it.

**Opinions about Episode 1: Representation biases and discrimination in healthcare**

Question 1 (i.e., about Episode 1: “I will think of bodies outside the norm”) was decomposed into 2322 segments. Based on the HCPC, five different clusters were identified. The first cluster (42% of segments / 82% of the students; called “Fatphobia in medicine”) indicated that students “realized” (v=6.6) the amount of “fatphobia” (v=6.3) and “prejudices” (v=3.6) towards “obese” (v=9.6) or “overweight” (v=5.7) “persons” (v=9.6) in the society in general, and especially in the healthcare field. This was a shocking realization (“shocked” [v= 4.8]) for some of them and sometimes “reminded” (v=3.4) them of situations that they had experienced themselves in the hospital.

The second cluster (23% of segments / 67% of the students; called “Considering weight issues in clinical practice”) discussed the interaction between a physician and a patient with obesity. The students highlighted that they ~~learnt~~ **learned** about how to better address the weight issue during a consultation (“how” [v=9.4], “address” [v=13.4], “subject” [v=11.0], “weight” [v=9.4]) and that they would not hesitate to ask the patient's help for the clinical examination if needed (“hesitate” [v=8.5], “help” [v=9.4.], “examination” [v=6.4]). The students indicated that they would integrate these suggestions in their future practice (“future” [v=7.1], “practice” [v=7.7]).

The third cluster (23% of segments / 64% of the students; called “How to tackle discrimination”) was about the harmful consequences of discrimination on the quality of care and ways of tackling this issue. The students highlighted that discrimination induces a loss of opportunity to receive adequate care (“loss” [v=5.2], “opportunity” [v=3.9], “delay” [v=7.2], “mistake” [v=6.7]) and that options to tackle this issue include creating a trusting, empathic and inclusive relationship, and cultivating a therapeutic alliance (“empathy” [v=5.4], “trust” [v=7.3]).

The fourth cluster (10% of segments / 41% of the students; called “Change about representation bias”) described how listening to this episode had raised awareness about representation bias and the normative body in medicine, or reinforced awareness of this issue in others (“change” [v=8.8], “idea” [v=12.0], “representation” [v=25.9], “bias” [v=20.4], “normative” [v=17.5], “body” [v=14.6], “awareness” [v=7.4]). Students emphasized the power of “testimonies” (v=5.5) to achieve this awareness.

The fifth cluster (2% of segments / 7% of the students; called “Psychological factors of obesity”) addressed the role of psychological factors in triggering or perpetuating obesity (“psychological” [v=10.1], “trauma” [v=13.1], “origin” [v=6.4], “cause” [v=4.7]). More specifically, students became more aware that it is important to consider psychological trauma as one of the causative factors of obesity. They also realized that it is critical to avoid judgement about patients with obesity as it can worsen eating disorders and create additional health problems.

**Opinions about Episode 2: Solidarity among healthcare professionals**

Question 2 (i.e., about Episode 2: “I will cultivate a benevolent and inclusive solidarity”) was decomposed into 2161 segments. The first cluster (48% of segments / 82% of the students; called “Common traditional culture at French medical schools”) discussed the role of the historic/conventional culture (which in French is called the “culture carabine”, a term alluding among other aspects, to a sexist, male-dominated environment at the hospital) in fostering solidarity among students of the medical school and medical doctors (“culture” [v=11.3], “carabine” [v=10.7], “tradition” [v=5.7]). Contrasting opinions emerged. Most students appreciated that the culture was on the cusp of change by eliminating the discriminative component - especially sexist stereotypes - to become more respectful and inclusive (“sexist” [v=7.9], “racist” [v=5.0], “violence” [v=4.9], “evolve” [v=3.1], “inclusive” [v=4.7]), while a minority of students regretted that this culture would be lost.

The second cluster (29% of segments / 63% of the students; called “Solidarity and quality of care”) indicated that listening to this episode made the students realize the importance of solidarity and team spirit among healthcare providers in order to ensure good quality of care (“solidarity” [v=10.7], “importance” [v=9.2], “improved” [v=10.2], “quality” [v=6.9], “care” [v=16.6]).

The third cluster (23% of segments / 42% of the students; called “Solidarity during the Covid-19 crisis”) highlighted that times of crisis (“period” [v=12.6], “crisis” [v=23.2]) are associated with an increased “solidarity” (v=11.2) among “healthcare professionals” (v=9.4), as illustrated during the Covid-19 pandemic [“covid” v=20.0].

**Opinions about Episode 3: Consent to healthcare**

Question 3 (i.e., about Episode 3: ”I will seek consent actively and at each instant”) was decomposed into 2144 segments. The first cluster (52% of segments / 91% of the students; called “Consent to healthcare: what and how”) indicated that students learned what consent to healthcare is, and how to obtain it in clinical practice (“ask” [v=8.3], “consent” [v=4.4], “yes” [v=5.4], “no” [v=6.4]). They mentioned that consent should be “explicit” (v=4.2) and that obtaining consent to healthcare is an active, ongoing process (“any” [v=4.1], “time” [v=4.3]), critically depending on a relationship of trust between patient and doctor (“relationship” [v=5.9], “trust” [v=7.2]).

The second cluster (39% of segments / 78% of the students; called “Gynecological violence”) focused on the consent issue in the context of a gynecological examination (“gynecological” [v=10.9], “violence” [v=11.8]). Students found the episode of particular interest (“interesting” [v=6.9], “particularly” [v=6.2]), especially the “testimonies” (v=7.6). They insisted that progress had to be made in this area.

The third cluster (9% of segments / 34% of the students; called “Importance of consent to healthcare”) emphasized that listening to the episode made the students realize the importance of consent to healthcare in clinical practice (“change” [v=13.8], “idea” [v=14.9], “after” [v=11.5], “listening” [v=23.3], “importance” [v=11.5], “consent” [v=10.8]), which they were planning to take into account for the “future” (v=3.9).

**Opinions about Episode 4: Mental health of healthcare professionals**

Question 4 (i.e., about Episode 4: “I will take care of myself to take care of others”) was decomposed into 1296 segments. The first cluster (62% of segments / 90% of the students; called “Vulnerability of healthcare professionals”) highlighted the particular “vulnerability” [v=2.1] of healthcare professionals to psychological difficulties, because of the permanent contact with suffering, sickness, and death. This cluster also mentioned a “pressure” [v=3.9] to keep going, caused by the need to fit the image of the “doctor superhero” (highlighted by the distinction between “doctors” [v=2.3] and “humans” [v=2.32], even when they suffer from mental health problems). The cluster also mentioned the need to adapt work conditions and career paths more easily.

The second cluster (25% of segments / 75% of the students; called “Caring for yourself”) emphasized that listening to the episode (“listening” [v=12.7], “episode” [v=18.5], “after” [v=7.8]), and especially the “testimonies” [v=10.5], “touched” most students [v=7.2] and helped them to “realize” [v=6.8] that mental health issues were very common among medical students and healthcare professionals. They became aware that caring for themselves was crucial (a minority of them were already aware of this point). Interestingly, some students mentioned that they felt less alone/isolated with their psychological issues after listening to the episode.

The third cluster (13% of segments / 50% of the students; called “Asking for help”) underlined that, in their future practice (“future” [v=17.5], “practice” [v=16.8]), students would not be ashamed to ask for help (“ask” [v=17.5], “help” [v=18.0]) when experiencing mental health difficulties. They felt that they would pay special attention to any difficulties their “colleagues” might be experiencing (v=10.3).

**Opinions about Episode 5: Scientific truth**

Question 5 (i.e., about episode 5: “I will look a little further for the truth”) was decomposed into 1479 segments. The first cluster (48% of segments / 93% of the students; called “Continuous medical education”) reported that students were eager to remain well informed during their career (“remain” [v=6.1], “informed” [v=4.4], “future” [v=7.4], “practice” [v=8.5]), in order to be able to provide optimal care for their patients and respond appropriately to their questions (“best” [v=4.6], “care” [v=4.8], “question” [v=4.1]). It emphasized that seeking scientific information at the source and on verified websites (“source” [v=4.1], “verified” [v=3.6]) was important for critical thinking about available medical knowledge (“critical” [v=5.2], “spirit” [v=2.1]). Students also recognized the value of sharing the limits of their knowledge with their patients.

The second cluster (42% of segments / 86% of the students; called “Scientific truth during the Covid-19 crisis”) focused on how scientific truth was undermined during the Covid-19 pandemic – especially in the public debate - illustrating that it was inherently transient and fragile (“during” [v=9.2], “Covid-19” [v=12.0], “pandemic” [v=7.5], “crisis”[v=9.4], “scientific” [v=7.9], “truth” [v=6.8], “public” [v=3.4]), “debate” [v=3.5]). Most students found the episode “interesting” (v=6.1) while a minority of them reported being “already” (v=3.7) aware about that.

The third cluster (10% of segments / 35% of the students; called “Scientific truth and social networks”) highlighted that students “realized” [v=6.2] the critical influence of social networks on the transmission and dissemination of scientific information, with the risk of reading fake news and/or being trapped in a filter bubble (“social” [v=26.3], “network” [v=27.5], “fake” [v=17.6], “news” [v=17.4], “bubble” [v=8.2]).

**Opinions about Episode 6: populations with loss of healthcare opportunities**

Question 6 (i.e., about episode 6: “I will look after those I cannot see”) was decomposed into 2079 segments. The first cluster (36% of segments / 77% of the students; called “Care equity”) highlighted the need to make special efforts to provide appropriate care for disadvantaged or marginalized people. It also underlined that human contact is critical when dealing with individuals from these populations, who should be treated with respect and without judgement (“humanity” [v=3.6], “human” [v=9.5], “contact” [v=3.6], “trustful” [v=5.1] “relationship” [v=5.7], “treated” [v=5.1], “respect” [v=4.6], “judging” [v=5.2])

The second cluster (27% of segments / 77% of the students; called “Healthcare services in prison”) described how students learned how healthcare in French prisons can be difficult and shocking (“learn” [v=8.0], “discover” [v=8.2], “never” [v=8.5], “thought” [v=8.7], “care” [v=10.5], “French” [v=5.9], “prison” [v=5.8], “custodial” [v=7.6], “settings” [v=6.2], “shocked [v=6.2]).

The third cluster (23% of segments / 56% of the students; called “Loss of healthcare opportunity in prison”) highlighted that prisoners experience a loss of healthcare opportunities, especially regarding psychiatric disorders and chronic diseases (“psychiatric” [v=15.0], “chronic” [v=9.0], “disease” [v=11.9]). It also emphasized that custody *per se* is a significant “cause” (v=4.1) of morbidity (“addiction” [v=7.1]) and mortality (“suicide” [v=6.9]).

The fourth cluster (11% of segments / 43% of the students; called “Influence on future practice”) discussed how this episode could change students’ future practice (“future” [v=23.4], “practice” [v=23.1], “change” [v=12.1]). About half of the students considered that listening to this episode would help them in their future practice when taking care of various disadvantaged populations. In contrast, the other half did not think it would influence their future practice as they did not expect to find themselves in the position of providing healthcare to prisoners.

The fifth cluster (3% of segments / 13% of the students; called “New perspective of those overlooked by the care system”) indicated that after listening to this episode (“after” [v=11.1], “listening” [v=18.3], “episode” [v=13.0]) the students got a new perspective (“realize” [v=4.1], “change” [v=6.2], “aware” [v=4.3]) on the issue of persons left out of the healthcare system (“care” [v=6.6], “access” [v=8.9], “inequality” [v=6.7], “forgotten” [v=7.9]).
